# The Deposition of the Appendiceal Stump

**Published:** 1907-10

**Authors:** Benjamin Merrill Ricketts


### SELECTIONS AND ABSTRACTS.

### THE DEPOSITION OF THE APPENDICEAL STUMP

---

By Benjamin Merrill Ricketts, Ph. B. M., LL. D.

---

This subject is dealt with from two view points.

First.—The consideration of the various methods of deposing of the stump.

Second.—The causes of hemorrhage and its frequency.

In response to a number of circular letters to surgeons, sixty-four replies were received representing eighty thousand, eight hundred and forty-one appendectomies, with an average mortality of seven and half in acute and one three-quarters per cent. in the chronic cases.

Eighty per cent. of the total number of operations reported

---

Read before the Mississippi Valley Medical Association, October 9 1907 Columbus, O (Author's Abstract.)

have been done by the purse string method.

The number of acute and chronic cases are about equal in number.

The various per cents. of different techniques used, are given. Also the per cent. of cases in which the different ligatures and sutures of catgut and linen were used.

About sixty cases of hemorrhage from the stump are mentioned, forty of which are tabulated in detail, giving eleven deaths. Of the sixty cases of hemorrhage mentioned, all but one have resulted in the purse string methods. The technique of each of the sixty-four surgeons reporting is given briefly.

Investigation of this subject shows that many operators have and are abandoning the purse-string methods and that simple ligature for the firm and complete extirpation for the soft appendix, are the safer methods.

Any other than simple ligature and extirpation are absolutely condemned as the use of cat gut for ligature or suture is condemned.

There are five illustrations showing the vascular supply of the head of the cecum appendix and mesocolon and thirteen various forms of technique.

